# Calculating Retinal Contrast from Scene Content: A Program

**DOI:** 10.3389/fpsyg.2017.02079

**Published:** 2018-01-17

**Authors:** John J. McCann, Vassilios Vonikakis

**Affiliations:** ^1^McCann Imaging, Arlington, MA, United States; ^2^Advanced Digital Sciences Center, Singapore, Singapore

**Keywords:** glare, calculate light on retina, human response to light, lightness, scene content, neural spatial processing, open source code:MATLAB, high dynamic range HDR

## Abstract

This paper describes a computer program for calculating the contrast image on the human retina from an array of scene luminances. We used achromatic transparency targets and measured test target's luminances with meters. We used the CIE standard Glare Spread Function (GSF) to calculate the array of retinal contrast. This paper describes the CIE standard, the calculation and the analysis techniques comparing the calculated retinal image with observer data. The paper also describes in detail the techniques of accurate measurements of HDR scenes, conversion of measurements to input data arrays, calculation of the retinal image, including open source MATLAB code, pseudocolor visualization of HDR images that exceed the range of standard displays, and comparison of observed sensations with retinal stimuli.

## Introduction

Psychophysical experiments require measurements of the light coming from the scene to the observers' eyes. This data includes the luminance and the angular subtense of each scene element. High-Dynamic Range (HDR) scenes (McCann, 2007) introduce substantial amounts of intraocular veiling glare, resulting from the scattering of light inside the eye. Glare introduces a complex spatial transformation of scene luminances. The quanta catch of retinal receptors is the combination of scene luminances and optical distortions such as glare.

In order to model human response to light we need to understand the sequence of input stimuli at each stage in the visual pathway:

1. The light coming from objects (Array of luminances from the scene)
2. The light falling on the retina (Array of retinal contrast)
3. The light response of receptors (Rod and cone quanta catch)
4. The spatial processing in the visual pathway (Neural spatial comparisons)
5. The appearance reported by observers (Psychophysical measurements of sensations, or perceptions)

Often, psychophysical models compare scene luminance with observer data. Such models collapse four of the five stages listed above into a single black box mechanism. Studies of HDR scenes show that the second and fourth stages tend to cancel each other (McCann and Rizzi, 2012, p. 146–152). Namely, neural processing counteracts optical glare. In order to properly model neural processes leading to appearances, we must calculate the light imaged on the retina. That includes glare's spatial transformations of scene luminances as the input array for neural visual models.

This paper describes computer software that calculates retinal luminance images from scene luminance measurements. The software is based on the CIE Standard for Intraocular Glare (Vos and van den Berg, 1999), and makes specific adjustments for characteristics of the observers; namely, age and color of the iris. The software is implemented in MATLAB® and the code is freely available to all researchers^1^. The article describes the program and provides flowchart, source code, and links to test images, parameter files, and updates (http://mccannimaging.com/retinalContrast/code.html).

Scene Luminance, unambiguously defined in physics, is the name of the input array used by the Glare Spread Function (GSF) convolution in the MATLAB program. This paper uses the term Retinal Contrast as the name of the program's output image. The GSF convolution conserves the total energy in the input Scene Luminance array. It redistributes all of the energy in the input image into the output image. As described by Hecht et al. (1942) the light falling on receptors is attenuated by front surface reflection, intraocular and macular pigment absorptions. As well, the eyes' optics, pupil size, and curvature of the retina are not accounted for in our MATLAB program. This paper limits the term Retinal Contrast to be the specific output array of our MATLAB program. It is the normalized, linear photopic energy per pixel in a flat array congruent with the flat visual test targets. This term is intended to recognize the fact that this program models the spatial effects of glare, but does not model the human eyes' light attenuation and 3-D transformations. The program is designed as a tool to study the effects of the CIE Glare Spread function on measured scene luminances in HDR scenes. The program does not calculate retinal luminance.

It is extremely important to have accurate scene luminances for the input to the program. In addition, the paper describes techniques to generate and measure HDR scenes. Photographs, made with digital and silver halide cameras, are inaccurate records of scene luminance because of the cameras' optical veiling glare (McCann and Rizzi, 2007).

**Figure 1:**
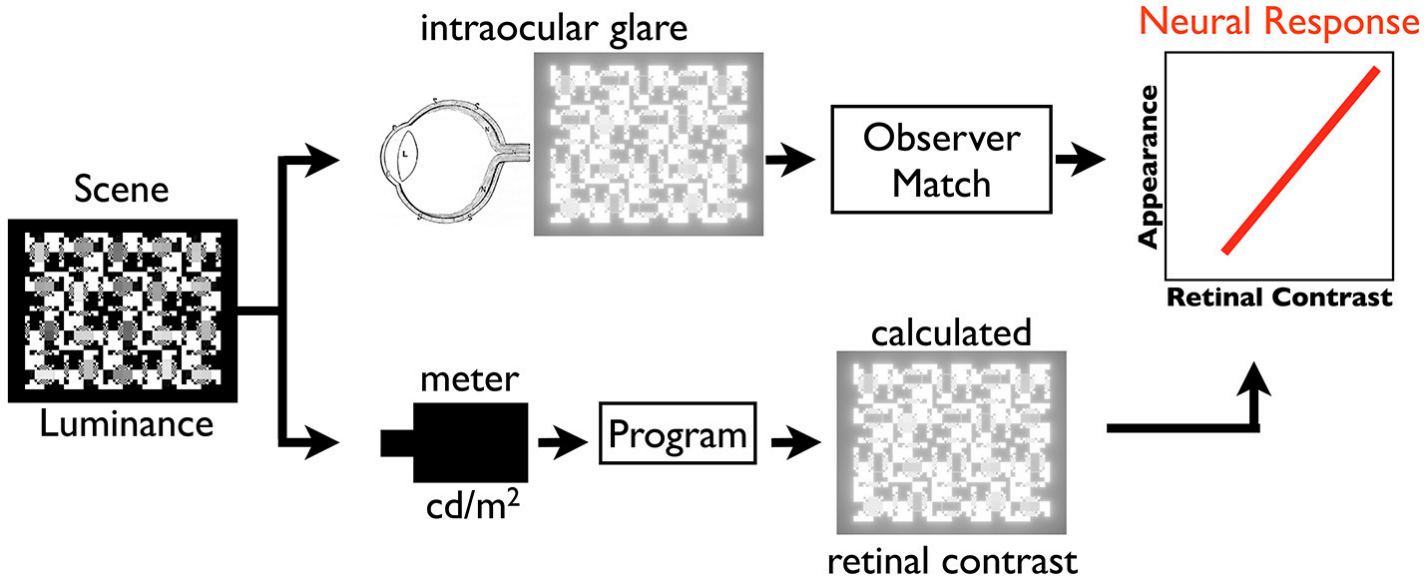
Illustration of the paper's technique to compare psychophysical appearance with retinal stimulus, calculated retinal contrast.

Figure 1 illustrates the paper's tools for studies of psychophysical models of vision. By converting scene luminance to relative retinal contrast, we can isolate the properties of neural spatial processing from optical veiling glare.

## Methods and materials: intraocular scatter

The influence of intraocular scatter can be found in two sets of psychophysical measurements. The first is the measurement by human observers of the standard Glare Spread Function (GSF). The second is the measurement of lightness sensations and their relationship to the image on the retina.

### CIE glare spread function (GSF)

Retinal straylight in normal vision varies due to age, and iris pigmentation. Further, straylight is the result of a variety of classes of cataracts, and straylight in the cornea. A comprehensive review can be found in “History of ocular straylight measurement: A review” (Franssen and Coppens, 2007), and “Ocular Media Clarity and Straylight” (van den Berg et al., 2010).

Retinal straylight is a visual handicap. Patient issues include hazy vision, contrast and color loss, difficulty with against-the-light face recognition, and halos around bright lights. Straylight will also adversely affect visual function tests, such as contrast sensitivity (van den Berg, 1986), visual field (van den Berg, 1987), and pattern electroretinogram (van den Berg and Boltjes, 1987).

Cobb (1911) introduced the concept of equivalent veiling luminance (*L*_*eq*_) as a way to define retinal straylight. Disability glare/retinal straylight, as defined by the CIE, is now quantified by means of this concept of equivalent luminance, i.e., the (external) luminance that has the same visual effect as the glare source at some angular distance (Vos, 1984; van den Berg and IJspeert, 1991).

*L*_*eq*_ is the outer part of the retinal GSF. The GSF is normalized to unity by writing

(1) $$\begin{array}{r} GSF=L_{eq}/E_{bl}\left(sr^{-1}\right) \end{array}$$

with E_bl_ the illuminance on the eye from the (glare) point source.

Tom van den Berg and J. K. IJspeert described a compensation technique to measure *L*_*eq*_/*E*_*bl*_ (van den Berg and IJspeert, 1991, 1992). Vos, chair of CIE TC1-18 (Disability Glare) and van den Berg developed a Standard Glare Observer (Vos and van den Berg, 1999) accepted as CIE standard (van den Berg, 1991; Vos et al., 2002). Vos and van den Berg (1999) provided a detailed description of the shape of the retinal PSF in their 1999 “Report on disability glare.” It includes an equation with parameters for age and eye color. Using this retinal PSF one can calculate the retinal image of any well-measured scene.

### Veiling glare accounts for lightness

Lightness is the visual appearance between white and black. Both psychophysics and neurophysiology experiments have measured the shape of the function describing response to light. The problem is that neurophysiology measures a logarithmic response, while psychophysics measures a cube-root function. That means that neurophysiology and psychophysics measurements give similar, but different, responses to light.

The magnitude of change in photocurrent from rods and cones is proportional to the logarithm of their quanta catch (Oyster, 1999). Many measurements of the logarithmic retinal responses have been reported (Hartline and Graham, 1932; Werblin and Dowling, 1969).

Using psychophysical measurements, the apparent Lightness scale in the Munsell Book of Color, L^*^ in CIELAB, and in CIELUV color spaces are fit by a cube-root of luminance (Wyszecki and Stiles, 1982, p. 486–513). Stiehl et al. (1983) made a complex lightness display using Munsell's lightness bisection technique. The display was made up of equally-spaced steps in lightness appearance. They measured the light coming from these equally-spaced lightness steps. Stiehl et al.'s lightness data was fit by CIELAB L^*^ functions, even though Stiehl's transparent target covered 3 log_10_ units of dynamic range, much greater than the range of reflectances found in the Munsell book (McCann and Rizzi, 2008).

Stiehl et al. (1983) showed that scattered light changes the shape of the psychometric cube-root function to a log function. They used the calculated retinal image based on Vos et al.'s (1976) GSF to calculate the spatial distribution of the light on the retina after intraocular scatter. They calculated the reduced contrast of the darker areas caused by scattered light in their test target. The appearance of lightness is proportional to the receptor response. Intraocular glare resolves the discrepancy between psychophysical (cube-root) and physiological (log) response functions. Cube-root applies to the target, while log applies to the retinal image.

### Glare calculation—converting scene luminance to retinal contrast

There are three steps in determining the spatial distribution of the light falling on the retina.

1. Measure each pixel's luminance in an HDR target (section Measurement of HDR Scenes)
2. Convert measurements to input data using meter calibration data (section Measurement of HDR Scenes)
3. Calculate the spatial distribution of retinal image (sections Optical Glare Spread Function to Flow Chart)

#### Measurement of HDR scenes

It would be very convenient to record a real-life HDR scene with a camera, and to use the captured digital array as the record of scene luminances. Although convenient, that would be a terrible mistake. A camera has an optical system that has its own glare spread function (McCann and Rizzi, 2007, 2012, p. 99–160). Consequently, images captured by cameras, will inevitably contain substantial glare distortion.

RAW format digital files are useful for removing the standard camera signal processes, such as tone scale and chroma enhancements (McCann and Vonikakis, 2012; McCann et al., 2013; LibRAW, 2017). However, using RAW format files does not remove the impact of optical glare on the captured luminances. RAW format photographs of a low-dynamic-range beach scene show glare distorted pixel values for objects with darker reflectances (McCann, 2014). Even though the scene had low dynamic range, it was mostly sky and sand, with near maximal luminances. The nearly all-white content of the scene generated enough optical veiling glare to seriously distort camera response digits. Camera image digits were not proportional to ColorChecker® reflectances under uniform illumination in a low-dynamic-range scene. In order to calculate the retinal image of a complex scene, we must have an accurate array of scene luminances, and cameras have been shown not to be able to provide such level of accuracy (McCann, 2014).

**Figure 2:**
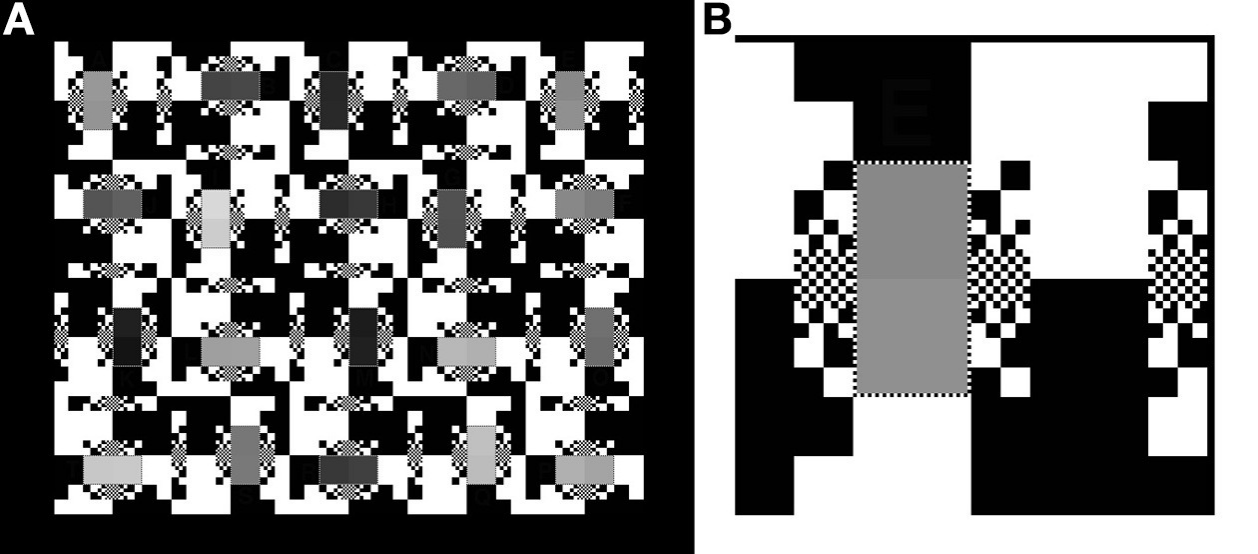
**(A)** Left: The “50% White” Background target. It subtends 15.5 by 19.1°. Its digital record (*inputMap* file) has 750 by 600 pixels. **(B)** Right: The 400% magnification of the upper right corner of “50% White” that illustrates the different sizes of the equal numbers of white and black squares making the background. The smallest, single pixel, white squares surround the pairs of gray test patches. The smallest pixel subtends 1.6 min of arc.

An optimal way to make a million-to-one dynamic range scene is to build it using transparencies. Photographic transparency film has a range of 3 log_10_ units. Two superimposed transparencies have a range of six log units. McCann and Rizzi (2012, p. 135–143) used pairs of photographic transparencies to fabricate HDR scenes with accurate luminances. Figure 2A shows an illustration of their achromatic HDR display made up of 20 pairs of test areas in a half white-half-black surround.

Figure 2B is a magnification of the upper right corner of the target. The 50% White background is made up of different size squares; ranging from 1 × 1 pixel to 64 × 64 pixels. The squares have the same distribution around all 20 pairs of test squares, although that pattern is rotated. This high contrast background was designed to have a middle average luminance (50% max) background. Further, this high contrast pattern was designed to have spatial contrast in many spatial frequency channels.

The Figure 2 test target was designed in Photoshop® as an achromatic 8-bit array. It is used as a 2-D digital map of the spatial locations of all the image segments. It is a digital paint-by-numbers map of the scene.

These input map digits were then used twice:

- First, the digital *inputMap* file was used as the file read by a film recorder that transformed digits 0–255 into film optical densities. The map array was printed by a laser scanner on 10 × 12.5 cm Ektachrome film.
- Second, the same *inputMap* integer array file was used in this MATLAB program to calculate floating point values for *sceneLuminance*. The film was measured with a transmission densitometer calibrated over 3.5 log_10_ units. Log luminances, or Optical Densities (OD), its reciprocal, are a data format that makes sense for HDR scenes. These measured optical densities are used to formulate a *conversionTable* to transform the 8-bit *inputMap* integers into a linear double precision *sceneLuminance* image. The two superimposed films generated the HDR scene for observers; and the calibration measurements made an accurate 6 log-unit range *sceneLuminance* input digital array for the intraocular glare calculation.

**Figure 3:**
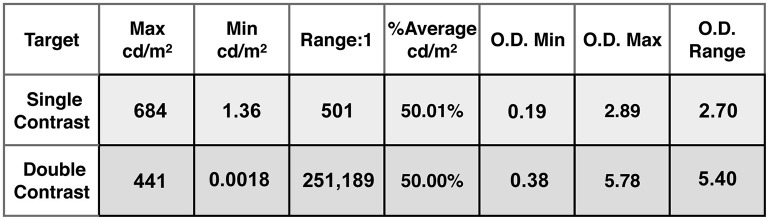
List of luminances, ranges, and average luminances of single and double contrast targets.

The film-recorder strategy allowed us to make a test target using Photoshop's integers for the *inputMap*. The maximum luminances of the Single and Double Contrast transparency targets were measured using a Konica Minolta Chroma meter CS-100A meter. The less than maxima luminances were from densitometer readings of film transparencies (single contrast), and their sum (double contrast). The measurements of optical density resulting from the 0 to 255 input values were accurate to 0.01 OD units over the film's 1,000 to 1 luminance range. The combined use of laser printed transparencies and film densitometer measurements, creates an accurate, high-precision luminance array covering 3 log_10_ units (single density). Two superimposed identical transparencies made the 6 log_10_ unit (double density) HDR displays. The luminances, Max, Min, Range, %Average luminances, and optical densities, and range measurements of both single and double density targets are shown in Figure 3.

It is interesting to note two properties of these transparency targets. First, the luminance range of test squares in Single Contrast is 501:1; and in Double Contrast is 251,189:1. That is 2.9 and 5.8 log_10_ units. Second, despite the squaring of the luminance range, the %Average luminance remains very close to constant. That is 50.01% in Single; and 50.00% in Double. The White background squares (50% of the background area) contribute almost all of the light to the average. The Black squares (50% of the background area) contribute one part in 500. By squaring the contrast range, the Black squares now contribute one part in 251,189. Those darker squares change the average by only 0.01%.

The transparencies were backlit by 4 diffused white light bulbs. The experiments were done in a dark room. The only source of light was the target. The lightbox had an average luminance of 1,056 cd/m2; with chromaticities *x* = 0.45, *y* = 0.43 (McCann and Rizzi, 2012, p. 136).

**Figure 4:**
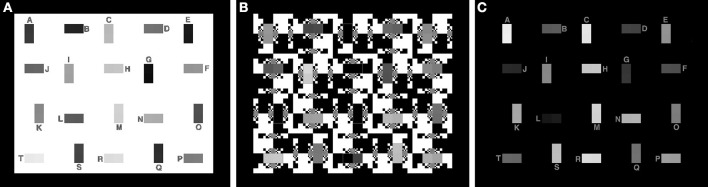
Test targets with nearly 6 log_10_ units of range with maximal, average and minimal glare. **(A)** 100% White background; **(B)** 50% White background; **(C)** 0% White background.

There are three test targets analyzed by this MATLAB program (Figure 4). In addition to the 50% White background shown in Figure 2, there are 100% White and 0% White for maximal and minimal glare.

#### Conversion of measurements to input data arrays

The MATLAB program converted measured luminances to the luminance input digit array. The MATLAB code is found in the Supplementary Material (Data Sheet 1).

- Reads the 8-bit integer *inputMap* Photoshop image used by the laser printer.
- Reads the 8-bit integer *conversionTable* log luminance calibration of the image.
- Calculates *sceneLuminance* linear data used to calculate retinal contrast. [8-bit digit-in is converted to measured double precision luminance-out].

The simple 0 to 255 *conversionTable* converts integer *inputMap* data into double precision *sceneLuminance* that accurately represents 6 log_10_ unit measurements with appropriate precision.

Although this process seems unusual, and a little complicated, it describes a technique for generating a complex HDR test target with reliable luminance values everywhere in the scene. The goal was to make a million-to-one dynamic range display with measured luminances. While HDR displays (Seetzen et al., 2004) using the combination of LED and LCD technologies have high dynamic ranges, the measurement of actual luminances (pixel by pixel) is a challenge. These displays send different spatial frequency signals to LED and LCD components. Laser recorder printed film is a simple, inexpensive, permanent media. When used in conjunction with small-spot densitometers it provides highly precise measurements of optical density. Superposition of two measured films makes a test image having a well-calibrated million-to-one range.

### Optical glare spread function

**Figure 5:**
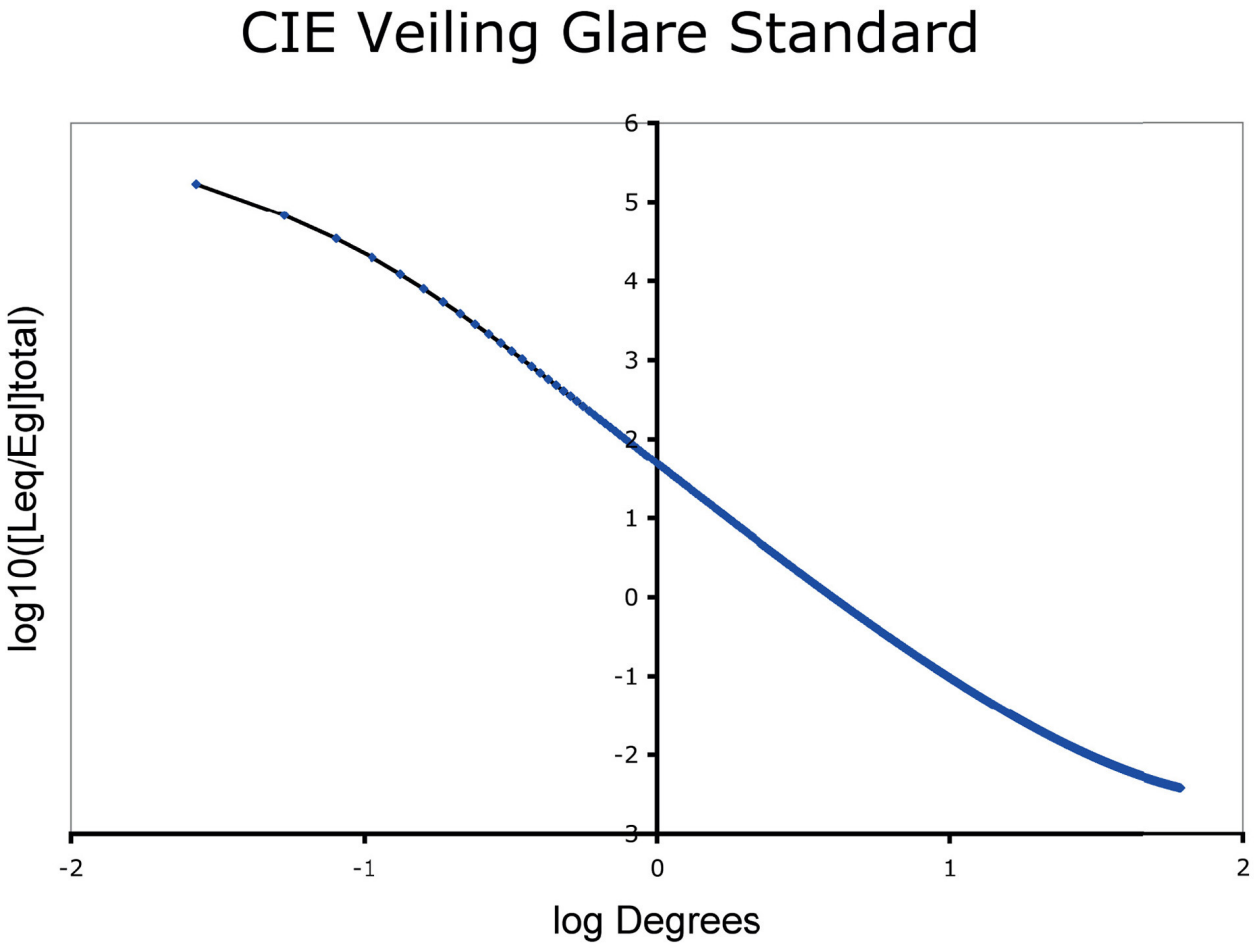
Glare Spread Function plotted on log-log axes.

The next operation used the GSF filter Equation (8) formula (Vos and van den Berg, 1999) to calculate the spatial distribution of the light on the retina. The CIE standard for veiling glare covers angles from 1/100 to 100 degrees (horizontal axis, Figure 5). The dynamic range of veiling glare (vertical axis) in the standard covers 1,000,000 to 1/1,000 units of the ratio of (equivalent luminance in cd/m2/glare illuminance at the eye in lux).

The retinal image is the sum of scene luminance, plus light scattered into each pixel. The amount scattered into each pixel is the sum of the veiling glare from all other pixels. Each glare contribution depends on the luminance of the distant pixel and its angular separation between the scattering and receiving pixels.

The CIE GSF is shown in Equation (2). We calculate the relative luminance at each pixel (*L*_*eq*_/*E*_*gl*_). It is the ratio of Equivalent Veiling Luminance (*L*_*eq*_ in cd/m2) and Glare Illuminance at the Eye (*E*_*gl*_ lux).

(2) $$\begin{array}{l} L_{eq}/E_{gl}=\left[1-0.08^{*}{\left(A/70\right)}^{4}\right]*\\ \left[\frac{9.2*10^{6}}{{\left[1+{\left(\theta /0.046\right)}^{2}\right]}^{1.5}}+\frac{1.5*10^{5}}{{\left[1+{\left(\theta /0.045\right)}^{2}\right]}^{1.5}}\right]\\ +\left[1+1.6^{*}{\left(A/70\right)}^{4}\right]*\{\left[\frac{400}{1+{\left(\theta /0.1\right)}^{2}}+3*10^{-8}*\theta^{2}\right]\\ +p\left[\frac{1300}{{\left[1+{\left(\theta /0.1\right)}^{2}\right]}^{1.5}}+\frac{0.8}{{\left[1+{\left(\theta /0.1\right)}^{2}\right]}^{0.5}}\right]\text{​}\}+2.5*10^{-3}*p \end{array}$$

where θ is the visual angle between emitting and receiving pixels, A is the age of the observer and *p* is the observer's iris pigmentation. This formula measures the equivalent veiling glare in relation to the energy of relative illuminance. Pigmentation types determine parameter values that range from 0 to 1.2. [*p* = 0 for very dark eyes, *p* = 0.5 for brown eyes, *p* = 1.0 for blue-green caucasians, up to *p* = 1.2 for blue eyes (Vos and van den Berg, 1999)]. In the calculations we used brown eye color *pigment* = 0.5 and *age* = 25.

### Glare spread function convolution filter kernel

Our retinal contrast calculation is the result of one of the many transforms performed by the optics of the eye. The Vos and van den Berg model provides the glare spread function that calculates glare as a function of visual angle. Our approach calculates the relative intensity on a plane, rather than on a sphere. It does not include other properties of the actual retinal image. For example, it does not incorporate the spatial transforms caused by the curvature of the retina. As well, it does not calculate the absolute photon count on the retina. Our paper calculates the relative retinal image contrast of the original scene as predicted by the 1999 CIE standard veiling GSF. The analysis compares two congruent digital arrays: the measurements of scene luminances with the calculations of retinal image contrast. We do not describe this array as retinal luminance, or retinal image because of the lack of absolute values, and geometric differences. We use the term *retinalContrast*.

**Figure 6:**
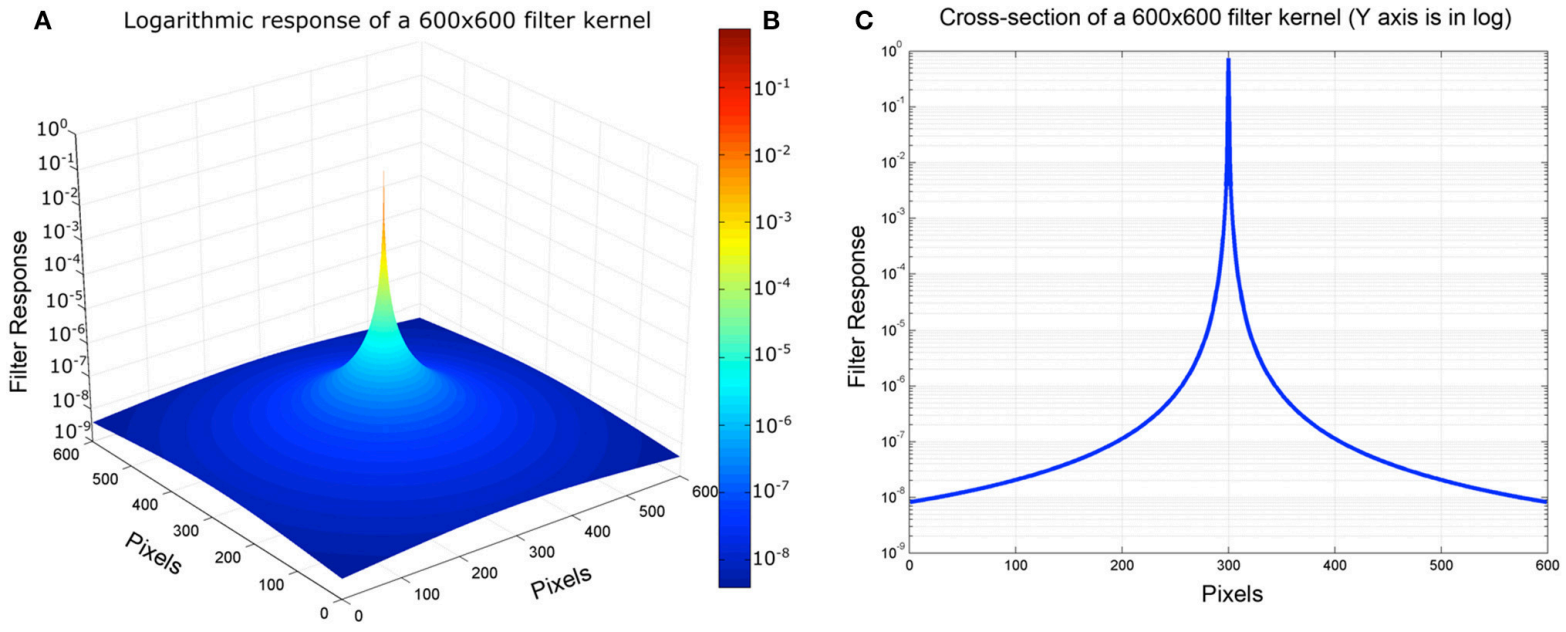
**(A)** Pseudocolor 3D plot of convolution kernel. **(B)** Color map illustration of log luminance kernel. **(C)** Cross-section of Log Luminance kernel.

Starting from the CIE GSF (Equation 2) we first compute the 2D filter kernel, which will be used in the convolution with the retinal input. The radius of the kernel is double the maximum size of the retinal input array, so it adjusts to the input dimensions. Even though the radius of the kernel is large, its values are never zero. This means that every position in the retinal input array will contribute to all the others, after the convolution with the 2D filter kernel. Once the values of the 2D filter kernel are calculated from Equation (2), they are normalized by their total sum, ensuring that they all add up to unity and thus, no DC constant is introduced during the convolution operation. Figure 6A depicts the 3-dimensional, and Figure 5C depicts the 2-dimensional plots of a 600 × 600 filter kernel. Note that, for visualization purposes, the output dimension is depicted in a logarithmic color scale (Figure 6B). However, during the actual convolution, the linear values are used. As it is visible from the two graphs, convolving the GSF kernel with the array of measured targets luminances, results in spreading the light from each pixel, into all other image pixels. The result for each pixel is the sum of a large contribution for the pixel's luminance, a few large contributions from nearby pixels and a very large number of very small contributions from distant pixels, since none of the kernel values are actually zero (Figure 6). Also, there is no radial distance at which the glare contribution reaches a constant asymptotic value.

The next operation computes the retinal image by convolving the filter kernel on the input luminance array, resulting in the calculation of the retinal contrast after the intraocular glare.

Performing the actual convolution, with such a large size kernel in the spatial domain, is very computationally expensive, since each of N pixels is affected by all others. As such, the complexity of this operation is O(N^2^). A typical approach for speeding up the computation is to perform the convolution in the frequency domain. This results in a O(NlogN) complexity. In our implementation we used the *imfilter* MATLAB command, which performs the convolution in the frequency domain by employing the fast Fourier transform (FFT).

The calculation of the 2D filter kernel, as well as the convolution operation with the retinal input array, are implemented in the “*computeRetinalContrast.m*” function of the provided MATLAB script.

### Input/output ranges

The fundamental idea in the calculation of *retinalContrast* from *sceneLuminance* is modifying the dynamic range. There are three different aspects to managing range in this calculation.

First, glare redistributes a small fraction of light from all pixels to all other pixels. The largest sources of light are the highest luminance pixels; the largest recipients of light are the lowest luminance pixels. It follows that the input image must represent the entire range of scene luminances accurately. Camera photographs are not good enough. The construction of the scene, its measurement with meters, and the viewing conditions for observer matching are all essential components of making accurate scene input (section Scene Dependent Human Response Functions).

The computational precision of pixel values is the second range aspect. The GSF convolution uses double precision to calculate the result of all pixels' contributions and tiny accumulations of light. This need for precision includes the padding of external input boundaries in the convolution.

The third aspect of range is visualization of the input/output information. By definition HDR scenes exceed the range of light possible in the media we use to inspect them. We need to carefully document the data (in and out) of the calculation in an accessible format. We also need tools to visually inspect these images that exceed the range of display devices.

#### Input data range

As described above in section CIE Glare Spread Function (GSF), the input design uses a paint-by-numbers *mapInput* (integers) in combination with calibration *conversionTable* (log_10_ integers) to calculate linear double precision *retinalContrast* values. The use of transparent targets in a darkroom insures that there is no other light source in addition to that coming from the target itself. The logarithmic table (0DD) assigns the lowest input to −1.0E+2, a low value approaching zero (representing the opaque part of the target and the darkroom). The second digit in the *conversionTable* is −6.17E+00 is the logarithm of the darkest scene measurement. The table permits the use of near 0 opaque luminance values for the outside border of the target that will be used for padding in the convolution.

While this is useful in the calculation, visual analyss of the results needs to restrict the range of data to be displayed to the relevant ranges of target luminances. The program uses *parameter.range* as a fixed range of luminance for analysis. The user of the program chooses its value based on the calibration data, and the range of interest in the analysis.

#### Computational padding

During the convolution operation, computing the values near the borders of the input array requires special treatment, since part of the kernel goes out of the area of the input array. A typical approach to address this is to pad zeroes (or any other value) around the original input array. The choice of the padded values however is very important; if the padded values are very different from the actual content of the input array, artifacts will appear on the borders of the final output. In order to minimize the impact of the padded values, we used the “replicate” option of the *imfilter* command, which mirrors the boundary values of the input array to the padded area. As such, the difference between the added padded values and the actual input content is very small, reducing the chances of border artifacts.

Vos and van den Berg (1999) describes the shape of the GSF. That shape does not include the glare loss of light from every pixel. The filter kernel was normalized so that the sum of all output retinal contrasts equals the sum of all input scene luminances. Without this step, the sum of output could exceed the sum of input. The filter calculates the light distribution on projected on a sphere (CIE GSF); and the program converts that to the light projected a plane (input pixels = output pixels). It does not include the effects of pre-retinal light absorptions.

#### Analysis range

**Figure 7:**
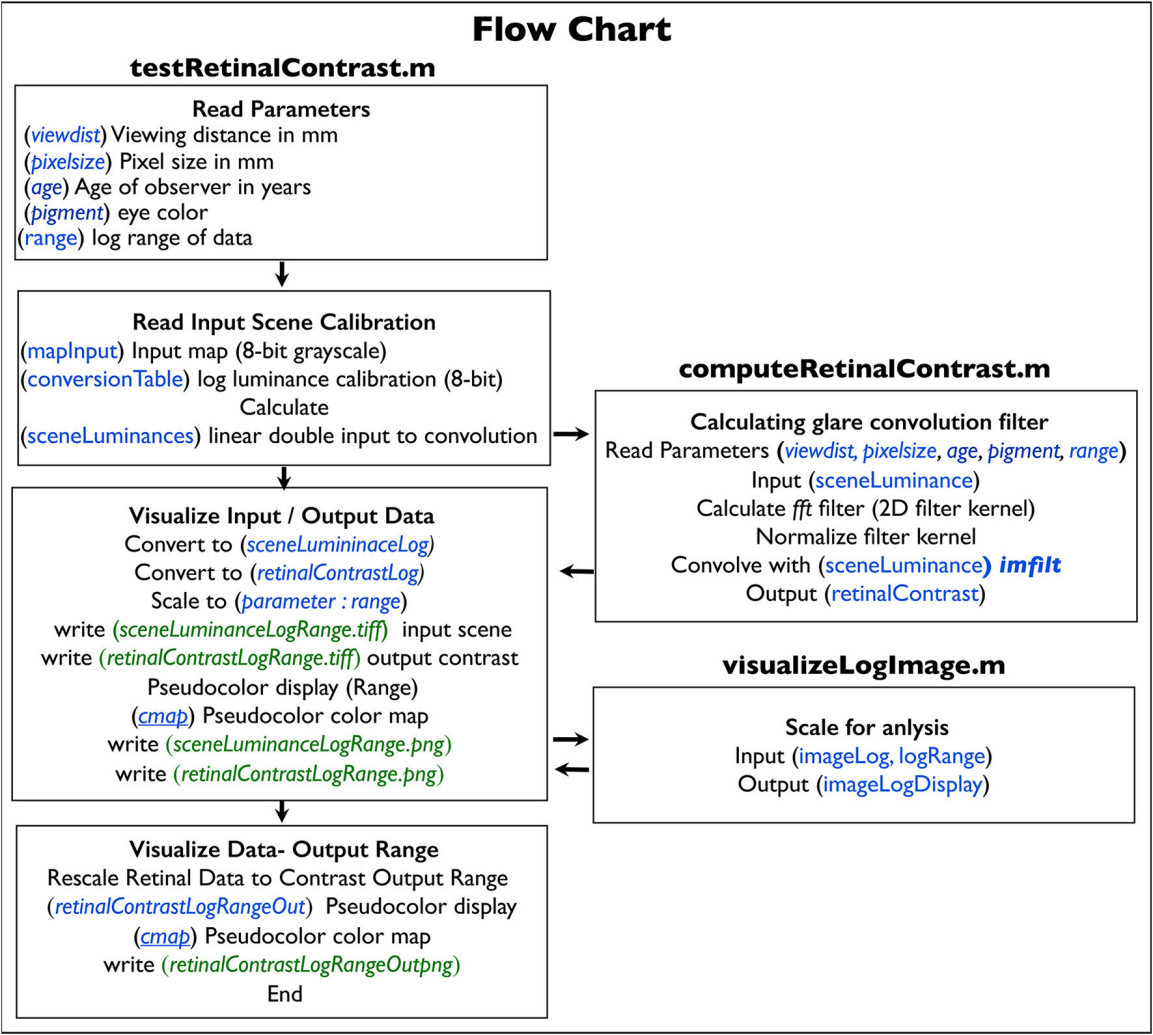
Flow chart of the program and functions.

The *testRetinalContrast.m* program (Figure 7) has input values between 0 and maximum luminance. For analysis, the program writes the output file *sceneLuminanceLogRange* (integer), which records the log luminance values scaled to *parameter.range*.

The output of the convolution is the *retinalContrast* array with linear, double precision values of the relative amounts of light on the retina. The content of the input scene, namely, the population and distribution of luminances determines the range in the *retinalContrast* output file. The greater the population of high-luminance pixels, the lower the *retinalContrast* dynamic range. As well, the local distribution of high-luminance pixels controls the local *retinalContrast*.

For comparison with input *sceneLuminanceLogRange*, the program writes the file *retinalContrastLogRange* (integer), that records the log values scaled to the same *parameter*.*range* value. This pair of files provides an accessible data format for numerical analysis of both input and output relative intensities using the same data scaling. In the HDR scene described above in Figure 2 *parameter.range* = 5.4. The range of analysis covers 5.4 log_10_ units.

The program saves the *sceneLuminanceLogRange.tiff*, and *retinalContrastLogRange.tiff* files, that are achromatic integers used to analyze numerical values of the retinal image. Visual inspection of this image is an unreliable tool for analyzing the spatial distribution of the light on the retina (McCann, 2017). Visual inspection does not represent the data they contain. Alternative analysis techniques are needed.

As an example, we can plot scans of digits converted to luminance across a horizontal window of input and output values. The plots are scans of the integers from *sceneLuminanceLogRange.tiff* and *retinalContrastLogRange.tiff*. These integers were scaled in the graph to the Log Relative Luminance range [−5.4, 0]. This is described below in section Pseudocolor “cmap.”

### Flow chart

As shown in the flow chart (Figure 7), the program *testRetinalContrast.m* reads input data for calculation arrays (blue text) and writes output files (green text). The program calculates the HDR *sceneLuminance* array used as input to the convolution. It calls function *computeRetinalContrast.m* that performs the convolution that calculates the retinal output array. It also calls the function *imageLogDisplay.m* that scales output data for analysis.

#### Mathematical formulation

Let matrix **M** ∈ ℤ^***w×h***^, denote the LDR input map that will be used to synthesize the scene luminance values, while *w* and *h* denote the width and height, respectively, of the map. The matrix **M** can be designed with any drawing software (e.g., Photoshop), and each of its elements *m*_*ij*_ takes values in the interval [0, 255], as a typical 8-bit grayscale image. In our software implementation, map **M** is represented by the *mapInput* array. Let function *C*(*x*):ℤ → ℝ that maps integer values to the domain of real numbers, based on linear luminance measurements taken from the actual scene. Function *C* essentially is a Look-Up-Table (LUT) containing scene luminance measurements, taken with a telephotometer. Since these measurements are in logarithmic scale, their values are raised to a power of 10 in order get the linear measurements. In our implementation, function *C* is implemented by the *conversionTable* array. The values of matrix **M** are mapped by function *C* in order to synthesize the actual scene luminance matrix **L** ∈ ℝ^**w×h**^ as follows:

(3) $$\begin{array}{r} \text{L}=C\left(\text{M}\right) \end{array}$$

Matrix **M** essentially provides the spatial patterns of the scene (or target), while function *C* provides the actual scene luminance values. In our implementation L is represented by the *sceneLuminances* array.

Let matrix **K** ∈ ℝ^(**2*r* + 1**)×(**2*r* + 1**)^ with *r* = *max*(*w, h*) denote a filter kernel representing the glare spread function as expressed by Equation (2). In order for the kernel **K** to be used for a convolution operation, it needs to be normalized so as to sum up to unity.

(4) $$\begin{array}{r} \tilde{\text{K}}=\frac{1}{\sum_{i,j=0}^{2r+1}k_{ij}}\text{K} \end{array}$$

where $\tilde{\text{K}}$ is the normalized glare kernel and *k*_*ij*_ the value of **K** at position (*i,j*). In our implementation kernel **K** is represented with the *filterKernel* array.

Finally, let matrix **R** ∈ ℝ^***w×h***^ represent the linear retinal contrast image which is derived by convolving the matrix **L** with the filter kernel $\tilde{\text{K}}$.

(5) $$\begin{array}{r} \text{R}=\text{L}*\tilde{\text{K}} \end{array}$$

where ^*^ denotes the convolution operator. In our implementation, matrix R is represented by the *retinalContrast* array, while the convolution operation is implemented in the frequency domain, using MATLAB's imfilter() function with replicating padding on the borders of the image.

## Results

The input and output of the GSF MATLAB code is a pair of integer arrays. The input array *sceneLuminanceLogRange*, and the output array *retinalContrastLogRange* cover the *parameter.range* log_10_ values. In this example, that range is 5.4 log_10_ units. The simplest analytical tool would be to use visual inspection to compare input and output. Such observations give the impression that glare has reduced the range, and the apparent sharpness of the retinal image. However, visual inspection of an array that represents 5.4 log_10_ units is extremely arbitrary and ambiguous. The variable *parameter.range*, and the calibration of the display, or printer, have control of the image under evaluation. Careful analysis of the input and output arrays require additional visualization techniques.

There are two techniques described here to analyze the HDR images.

- Plots of input luminances and output retinal contrast images (section Logarithmic Plots).
- Visualizations using pseudocolor colormaps (section Pseudocolor Analysis of Program Calculations).

### Logarithmic plots

**Figure 8:**
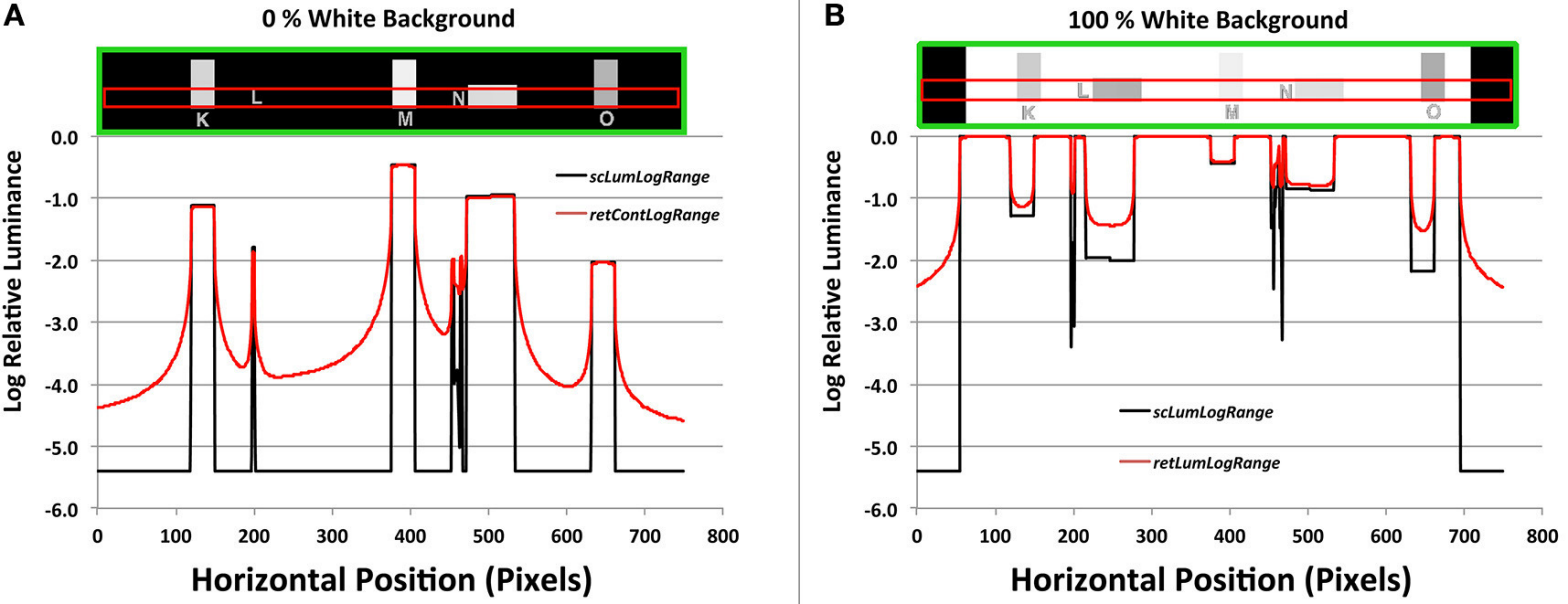
**(A)** Plots of a horizontal scan of the black (0% White) background, and **(B)** Plots of 100% White background targets. The green boxes at top show the horizontal target segments (Areas K,L,M,N,O); the red boxes show the scanned areas. The black lines in the graph plot input *sceneLuminancesLogScale*, and the red lines plot output *retinalContrastLogScale*.

The black background in 0% White (Figure 8A) is a million times lower luminance than the 100% White (Figure 8B). The only sources of glare in Figure 8A are the 40 test squares. They show tiny losses of light in their plots. That glare light from these squares replaces dark, uniform target areas with steep gradients; many change by more than a factor of 10. Depending on the position of the glare sources, the retinal image varies from 1/100's to 1/10,000's of the White's intensity.

All the test areas are darker than the background in 100%White target (Figure 8B). They show small increments in brighter squares, and larger increments in darker ones. Again, uniform input luminances become output gradients.

### Pseudocolor analysis of program calculations

Section Results describes, with examples, pseudocolor techniques for visualizing the *sceneLumuminanceLogRange* and *retinalContrastLogRange* data. Computer graphics helps to visualize the changes in scene luminance to retinal contrast. Pseudocolor renders intensity as a quantized, ordered set of colors. It breaks up apparently hard-to-see smooth gradients into clearly segmented color bands. By matching the color in the output array to the calibration scale, the reader can identify the amount of light (McCann and Rizzi, 2009).

The linear double-precision *sceneLuminance* and *retinalContrast* data are not suitable for pseudocolor rendering, because of their range. For example, consider using 64 color bins (4 digits wide) on the linear 5.4 log_10_ unit luminance input (range = 251,188: 1). The lowest bin includes a range of 3.0 log_10_ units. By converting the file from linear to logarithmic, every 4-digit bin represents the same ratio range of 1.05: 1. The program uses pseudocolor rendering for *sceneLuminanceLogRange*.*tiff* and *retinalContrastLogRange.tiff*.

### Pseudocolor “cmap”

**Figure 9:**
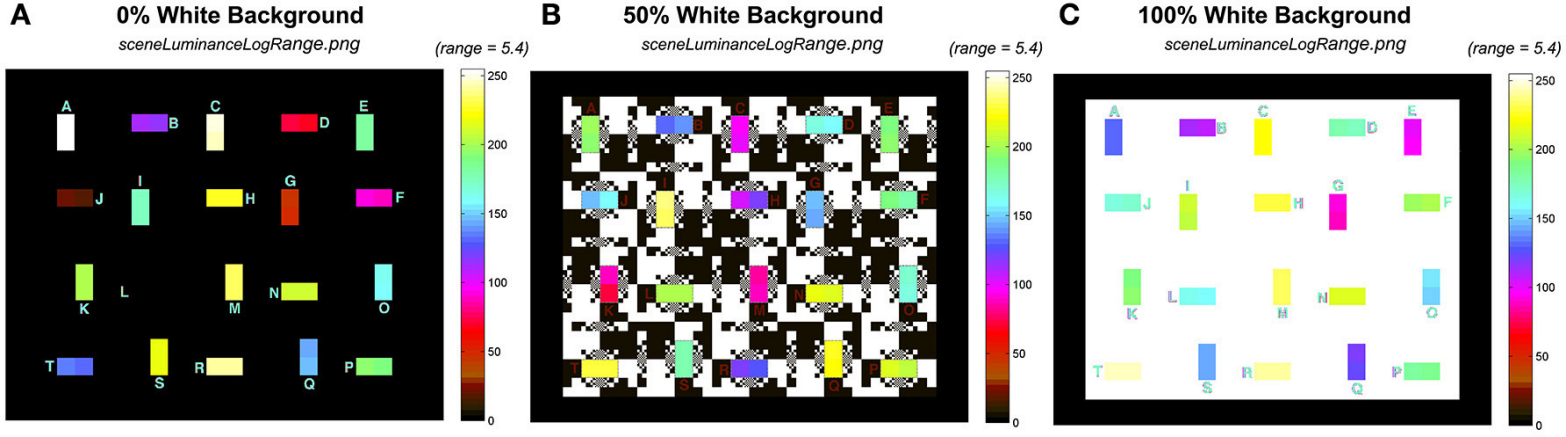
Comparison of the input *sceneLuminanceLogRange* calculation using the same 5.4 log unit pseudocolor range. **(A)** 0% White; **(B)** 50%, White; and **(C)** 100% White backgrounds.

The pseudocolor *cmap* has 64 color values, arranged in 8 progressions (See color scales in Figure 9 with corresponding digit values). Digit = 0 in is black. Each of the 64 colors has a bin width of 4 digits. The first 8-bin progression has 8 different color values starting at black, and ending at dark brown. The next three 8 color progressions end at red, magenta, and blue. Blue is the midpoint at digit 128, rendering relative log10 luminance = −2.7. The next four segments end at cyan, green, yellow, white, at log10 luminance = 0.0, or 100%.

### Input/output pseudocolor comparison

Figure 9 shows the pseudocolor rendition of input *sceneLuminanceLogRange.png* for 0%, 50%, and 100% White backgrounds. The range is 5.4 log_10_ units, for all three test targets with different backgrounds.

In Figure 9, all three input targets had a range of luminances close to 6 log_10_ units, covering most of the color scale. The 20 pairs of test squares had similar radiances in the test squares, but they were arranged in different locations. The changes in location for a particular luminance pair was designed to prevent observer memory location bias in apparent lightness measurements. The background was either max, or min luminances, with input edge ratios of almost 1 million to one.

**Figure 10:**
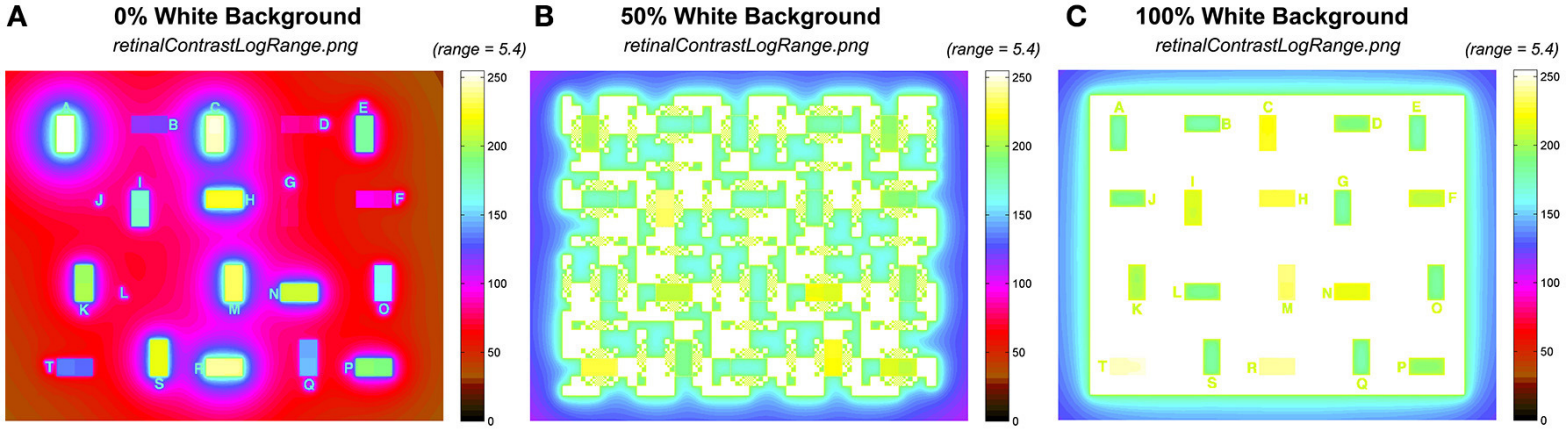
Comparison of the output *retinalContrastLogRange* calculations using the same 5.4 log_10_ unit pseudocolor range. **(A)** 0% White; **(B)** 50%, White; and **(C)** 100% White backgrounds.

Figure 10 shows the corresponding pseudocolor rendition of output *retinalContrastLogRange.png* for 0, 50, and 100% White backgrounds. The output range is reduced depending on the contents of the input scene.

Figure 10A shows the black background (0% White). It shows that the test squares spread non-uniform gradients of light that increase the background (−5.4) by 1–2 log_10_ units. Figure 10B shows the half-white /half black background (50% White). It shows that the max part of the background spreads more than 3 log_10_ units of glare into the largest minimum background areas. The effect of glare is greater in smaller minimum background squares. Further, Figure 10C with 100% White shows that the range of all test squares is compressed into <2 log_10_ units. In all three scenes the opaque outermost surround (section Input Data Range) varies substantially with scene content.

### Pseudocolor scaled to output

The dynamic range of test squares in the input *sceneLuminance* is close to 6 log units, while the output is variable with the content of the scene. The output files in *retinalContrastLogRangeOut.png* are scaled to the range of output, rather than input. The program finds min and max of *retinalContrastLogRange* and reassigns them digits 0 and 255. It rescales all digits to this variable range in *retinalContrastLogRangeOut* using *cmap*.

**Figure 11:**
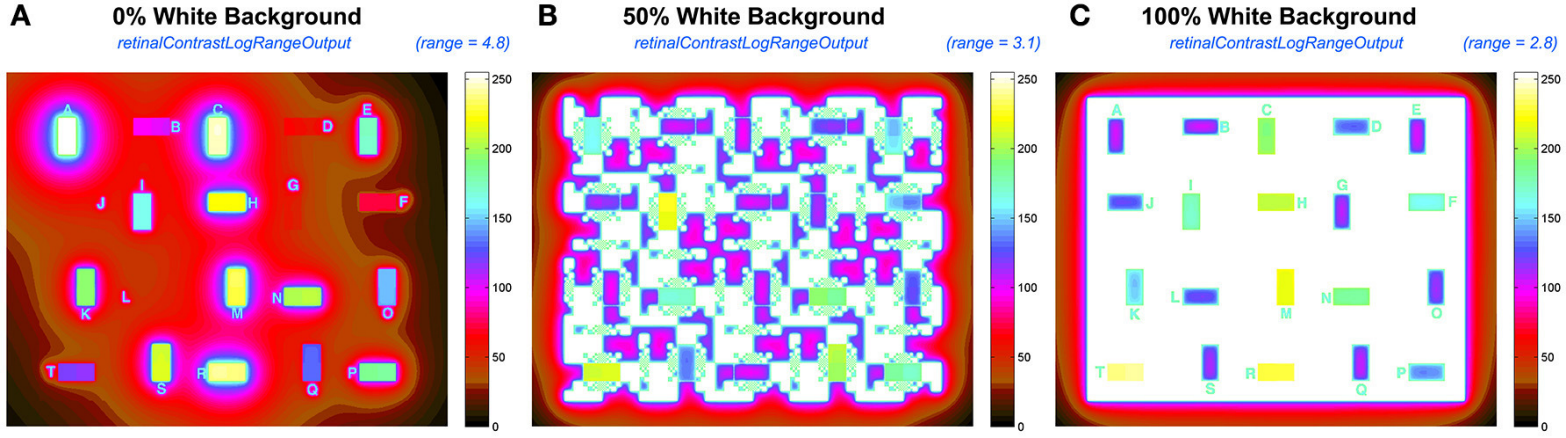
Comparisons of the output *retContLogRangeOut.png* renditions using variable pseudocolor ranges for each test target: **(A)** 4.7 log_10_ range for 0% White; **(B)** 3.1 log_10_ range for 50% White; **(C)** 2.8 log_10_ range for 100% White background.

By adjusting the range of the pseudocolor rendering, Figure 11 presents a more effective pseudocolor image. It provides a better tool for analyzing glare.

Figure 11 rescales the *retinalContrast* data using the entire *mapInput* array including the opaque border. In 0% White the darkest output pixel is 4.8 log_10_ units lower than maximum output. In 50% White that range is 3.1; and in 100% White is 2.8. The 40 test squares in the target have smaller ranges. The output range for test squares in *retinalContrast* varies from 4.0 log units for 0% White surround; while it is 1.5 log units for 100% White surround.

The highly variable range of light on the retina results from the content of the scene, namely, the spatial population of luminances, that controls the sum, and distribution of intraocular glare. The wide range of input luminances, and the variable range of outputs requires that the program user have interactive control of the pseudocolor's display's range. The program has that feature.

## Discussion

Models of human vision require accurate measurements of the field of view of the human observer as the input to the model. That input requires measurements of the visual angle subtended by objects in the field of view. As well, the input requires measurements to the luminances and radiances falling on the observer's eyes.

CIE Colorimetry Standards provide an interesting example. Color matching began with Maxwell (1860) as a technique to measure human response to light. Two adjacent lights are adjusted to match. At match the observer sees a single spot with fixed angle, on a no-light surround. The CIE Color Matching Functions (Wyszecki and Stiles, 1982, p. 124) generate very different spectral responses compared with absorption spectra of cone visual pigments. The reconciliation of these different measurements relies heavily on the transmission spectra of intraocular media (Smith and Pokorny, 1975).

Colorimetry and HDR images are extreme examples of studies of vision. Colorimetry uses a single spot that is the only source of intraocular glare. Colorimetry incorporates the optical transmission of ocular media as an essential component of its calculation. HDR scenes introduce ranges of light that are substantially transformed by intraocular media. Here the transformation is spatial, as well as spectral.

Again, in HDR imaging the optical transformations are necessary to reconcile the differences between psychophysical observation and neurophysiological measurements. CIE Lightness is a cube-root function of scene luminance, but a logarithmic function of retinal luminance. More simply, lightness is proportional to receptor response to quanta catch (see section Veiling Glare Accounts for Lightness)

### Human response to light

One of the first topics in the foundation of psychophysics in the 1860's was the measurement of the human response function to light. Fechner (1860) and Weber initiated the field by measuring the amounts of light that caused different visual sensations. This idea that light on the retina causes appearance is a cornerstone of human vision, at least in a broad general manner. Further, a second idea is broadly held: namely a photograph reproduces the light from the original scene on the retina.

Research in vision and photography over the past 150 years has refined our understanding of these generalizations. Studies of both adaptation of photoreceptor sensitivity (Dowling, 1978), and the important role of spatial neural interactions (McCann and Rizzi, 2012) have shown that the quanta catch of a single photoreceptor does not generate a unique sensation. As well, reproduction of a sensation is not uniquely generated by a fixed radiance. And, photographs do not reproduce scenes.

**Figure 12:**
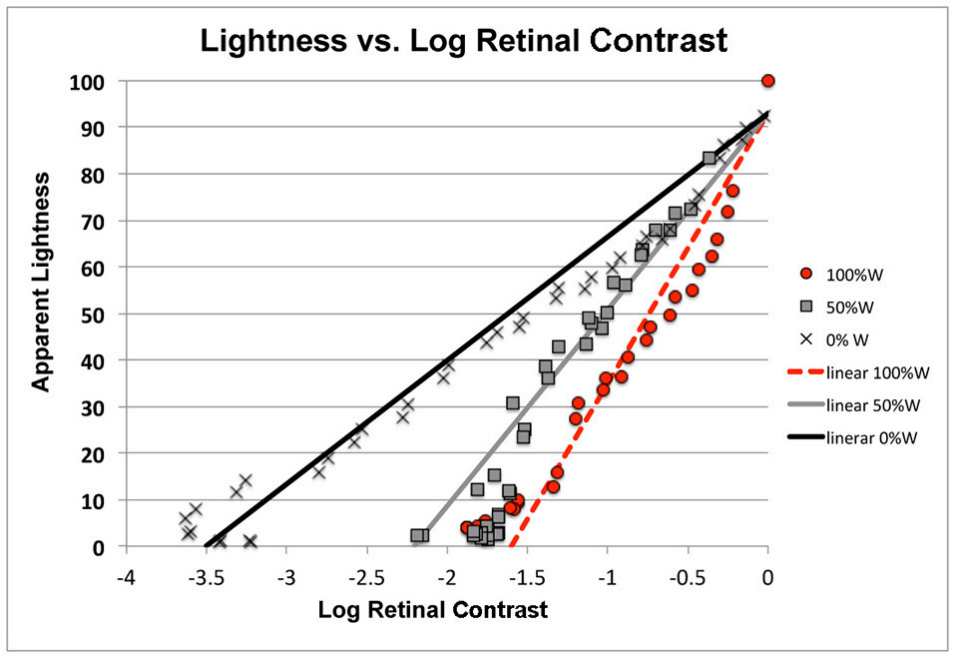
Plots of lightness sensations as a function of log retinal contrast.

Here we want to characterize the Human Response Function (HRF) to HDR stimuli. Rizzi and McCann (2009) measured sensations of lightnesses of the 40 test squares in the 0, 50, and 100% White test targets described above (See Figure 4). Figure 12 plots apparent lightness (psychophysical metric) vs. calculated log retinal contrast (physical metric) for these three targets. The retinal contrast values are calculated using the code included below.

We do not find a single function for observers' response to retinal contrast. Instead, we find three distinct responses, one for each background with different spatial contents. All three are linear functions of log retinal contrast. Lightness of all three targets is linearly proportional to receptor response. However, Figure 12 shows the content of the scene on the retina initiated very different amplification slopes of receptor response to quanta catch.

Figure 12 plots the fit by three linear functions of log retinal contrast. They are three independent measures of the Human Response Function to light. The values of slope (m) and intercept (b) are listed in Table 1.

**Table 1:** Slopes, intercept, and correlation coefficients of the different linear HRFs for each background.

**Background**	**Slope (m)**	**Intercept (b)**	**Correlation**
White	56.3	93	0.93
White/Black	47.0	93	0.92
Black	26.7	93	0.97

Apparent lightness is a logarithmic function of quanta catch, and a linear function of receptor response (Oyster, 1999). However, the slope of that appearance plot varies with the content of the scene. A 100% White background causes the highest glare, and therefore has the lowest contrast retinal image. Nevertheless, it has the highest apparent contrast. The slope of that human response function is 56.3. The Black background has the least glare, yet the human response function has the lowest slope of 26.7. With 0% White, it takes 3.5 log units of decrease in dynamic range to go from the sensation white to the sensation black. In comparison, that same white/black change in sensation is observed in 1.6 log_10_ units (100%White) with much more intraocular glare.

### Scene dependent human response functions

We can model the different Human Response Functions with a very simple equation.

(6) $$\begin{array}{r} L=\left(26.7+S\right)\log R+93 \end{array}$$

where *L* is apparent lightness (Scale 100:1); *R* is retinal contrast; and *s* is an additive factor responsive to scene content. In the three HDR scenes studied here, *s* = 0 for the Black surround; *s* = 20 for the half-White/half-Black background; and *s* = 30 for 100% White background. A small signal that adds to the slope that amplifies log retinal contrast can model lightness in HDR, and scenes in the real visual environment.

The implication of this equation is that the post-receptor visual processing is scene dependent. There is no single Human Response Function for all receptor quanta catches. The data require a dramatic change in the slope of the HRF with changes in scene content with constant dynamic range. The remaining problem is to define the model for calculating the parameter s from the spatial array of retinal radiances.

## Conclusions

Models of vision's response to HDR scenes have to go beyond simple, single-pixel responses to light. Vision has two powerful spatial processes that transform scene radiances. The first transform is the degradation of the optical image by glare, and the second is the enhancement by post-receptor neural mechanisms. A comprehensive model of vision requires both elements. The problem of calculating appearance is that these two strong mechanisms almost cancel each other. This has the advantage that we rarely notice glare in everyday life, but the disadvantage that it makes the separation of their properties more difficult for scientific analysis.

This paper describes a computer program that calculates the relative contrast on the human retina. It also provides the program's code based on the work of Vos and van den Berg (1999). In addition, it describes the important task of creating accurate scene luminance input data for the program. Although convenient, digital data from cameras is not accurate. Camera optical veiling glare distorts the image on cameras' sensors. Input data must be measured with telephotometers, or densitometers, to insure that the spatial record of input scene luminances is accurate.

The response of the eye to light depends on the spatial luminance content of the scene; and the glare-dependent consequential retinal image. The lightness appearance is proportional to retinal receptor response. However, post-receptor neural processing controls the slope of that appearance response function. The slope varies with the contents of the scene.

## Author contributions

JM and VV have collaborated in the preparation of open source MATLAB code for distribution; VV wrote and implemented the code and collaborated with JM in the analysis; JM has brought together this glare and lightness research in collaboration with many others.

### Conflict of interest statement

The authors declare that the research was conducted in the absence of any commercial or financial relationships that could be construed as a potential conflict of interest.

## Footnotes

^1^*Image Processing Toolbox*. Available online at: https://www.mathworks.com/products/image.html?s_tid=srchtitle

## Supplementary material

Datasheet 1 MATLAB code.
