# Supplementary material for: Calculating Retinal Contrast from Scene Content: A Program
Source: Front Psychol. 2018 Jan 17;8:2079. doi: 10.3389/fpsyg.2017.02079 (PMC5776149; doi:10.3389/fpsyg.2017.02079)
Supplement: Datasheet 1 — MATLAB code. [file DataSheet1.docx]

**Supplementary Data Sheet**

Calculating Retinal Contrast from Scene Content

Program Source Code

John J. McCann 1* and Vassilios Vonikakis 2

1 [mccanns@tiac.net](mailto:mccanns@tiac.net), 2 <bbonik@gmail.com>

Please visit our website for Source Code updates, sample images and new freely distributed versions. We are currently developing open-source Python code that will not need a commerical toolbox.

The update website is:

[**http://mccannimaging.com/retinalContrast/code.html**](http://mccannimaging.com/retinalContrast/code.html)

**Matlab Code** (version 1.0; Dec 1, 2017)

**%testRetinal Contrast.m**

% This script tests the retinal contrast function on scene luminances.

% First the parameters are set. Then the function computes input scene

% luminances by combining a 2D input map of the scene and a table of

% telephotometer readings of the scene. This is done because telephotometers

% are the only reliable method of estimating accurate luminances without

% being affected by the effects of glare.

%

% mapInput (unit8): 2D paint-by-numbers map of scene used to calculate luminance

% conversionTable (double): calibration measurements of scene luminances

clear all

close all

tic

%--------------------------------------------------------setting parameters

parameters.age=25; %age of the observer

parameters.pigmentationFactor = 0.5; %(p=0 for very dark eyes, p=0.5 for brown eyes, p=1.0 for blue-green %caucasians, up to p=1.2 for blue eyes).

parameters.pixelSize = 0.1664; %in mm

parameters.viewingDistance = 360; %in mm

parameters.verbose= true; % display detailed messages during runtime

parameters.range=5.4; % output range in log units

%----------------------------------------------------------Loading map file

inputMapFilename='0N_750x600.tiff'; %filename of the image to be processed

conversionTable = load('lglut-0DD.txt'); %filename of the spectrometer measurements of scene luminances

mapInput = imread(inputMapFilename);%load image file

if (size(mapInput,3)1)

mapInput=rgb2gray(mapInput); %if not grayscale, convert to grayscale

end

mapInput=imresize(mapInput,0.5); % half the size of input map (to speedup the execution)

%display original image

figure, imshow(mapInput);

colorbar

title('INPUT map');

%defining visualization map for pseudocolors

pseudocolors=[

0 0 0

0.0825 0.061875 0

0.165 0.12375 0

0.2475 0.1134375 0

0.33 0.103125 0

0.4125 0.07734375 0

0.495 0.0515625 0

0.5775 0.19078125 0

0.66 0.33 0

0.7012 0.28875 0

0.7424 0.2475 0

0.7836 0.20625 0

0.8248 0.165 0

0.866 0.12375 0

0.9072 0.0825 0

0.9484 0.04125 0

1 0 0

1 0 0.125

1 0 0.25

1 0 0.375

1 0 0.5

1 0 0.625

1 0 0.75

1 0 0.875

1 0 1

0.91625 0.04125 1

0.8325 0.0825 1

0.74875 0.12375 1

0.665 0.165 1

0.58125 0.20625 1

0.4975 0.2475 1

0.41375 0.28875 1

0.33 0.33 1

0.35125 0.41375 1

0.3725 0.4975 1

0.39375 0.58125 1

0.415 0.665 1

0.43625 0.74875 1

0.4575 0.8325 1

0.47875 0.91625 1

0.5 1 1

0.5 1 0.9375

0.5 1 0.875

0.5 1 0.8125

0.5 1 0.75

0.5 1 0.6875

0.5 1 0.625

0.5 1 0.5625

0.5 1 0.5

0.5625 1 0.4375

0.625 1 0.375

0.6875 1 0.3125

0.75 1 0.25

0.8125 1 0.19

0.875 1 0.13

0.925 1 0.06

1 1 0

1 1 0.125

1 1 0.25

1 1 0.375

1 1 0.5

1 1 0.625

1 1 0.75

1 1 0.88

1 1 1];

%----------------------------------------------Calculating scene luminances

%applying the conversion table on the scene map

conversionTable=10.^conversionTable;

sceneLuminance=conversionTable(mapInput+1);%taking care of Matlab's indexing [0,255]-[1,256]

%statistics of the scene luminance

maxSceneLuminance = max(max(sceneLuminance));

minSceneLuminance = min(min(sceneLuminance));

rangeSceneLuminance = maxSceneLuminance/minSceneLuminance;

meanSceneLuminance = mean(mean(sceneLuminance));

if (parameters.verbose)

fprintf('\n Scene luminance statistics');

fprintf(['\n max=' num2str(maxSceneLuminance)]);

fprintf(['\n min=' num2str(minSceneLuminance)]);

fprintf(['\n mean=' num2str(meanSceneLuminance)]);

fprintf(['\n range=' num2str(rangeSceneLuminance) '\n\n']);

end

sceneLuminance=(sceneLuminance./maxSceneLuminance);%normalizing scene luminance to the maximum

%----------------------------------------------------------estimating glare

%calling the glare estimation function 'computeRetinalContrast.m'

[retinalContrast]=computeRetinalContrast(sceneLuminance, parameters);

%-----------------------------------------Visualizations with and without pseudocolors

%--for scene luminance

sceneLuminanceLog=log10(sceneLuminance); %range=[-100,0]

sceneLuminanceLogRange=visualizeLogImage(sceneLuminanceLog, parameters.range);%apply the visualization range on the log image

imwrite(sceneLuminanceLogRange,'sceneLuminanceLogRange.tiff','tiff');% save the log image display log %scene luminance with pseudocolors

figure, imshow(sceneLuminanceLogRange);

colorbar

title('INPUT Scene Log Luminance Range');

colormap(pseudocolors);

print -painters -dpng -r300 sceneLuminanceLogRange.png

%--for retinal contrast

retinalContrastLog=log10(retinalContrast);

retinalContrastLogRange=visualizeLogImage(retinalContrastLog, parameters.range);%apply the visualization range on the log image

imwrite(retinalContrastLogRange,'retinalContrastLogRange.tiff','tiff');

%display log retinal contrast with pseudocolors

figure, imshow(retinalContrastLogRange);

colorbar

colormap(pseudocolors);

title(['Retinal Contrast Log Range = ' num2str(parameters.range)]);

print -painters -dpng -r300 retinalContrastLogRange.png

%--for retinal contrast (output range)

%Scaling pseudocolor retinal contrast image to output range (scene dependent)

%Glare reduces the range of output. The following code reduces the pseudocolor range to that of the output.

minRetinalContrastLogRangeOut = min(min(retinalContrastLog));

maxRetinalContrastLogRangeOut = max(max(retinalContrastLog));

rangeRetinalContrastLogRangeOut = maxRetinalContrastLogRangeOut-minRetinalContrastLogRangeOut;

retinalContrastLogRangeOut= 255.*((retinalContrastLog-minRetinalContrastLogRangeOut)./rangeRetinalContrastLogRangeOut);%rescale to output

retinalContrastLogRangeOut=uint8(retinalContrastLogRangeOut);

figure, imshow(retinalContrastLogRangeOut);

colorbar

colormap(pseudocolors);

title(['Retinal Contrast Log Range Output = ' num2str(rangeRetinalContrastLogRangeOut)]);

print -painters -dpng -r300 retinalContrastLogRangeOut.png

toc

**function [retinalContrast]= computeRetinalContrast(sceneLuminance, parameters)**

% RetinalContrast:

% Calculation of the array of light falling on the retina, based on Vos and van den Berg (1999) equations.

% The function uses the CIE standard glare spread function in order to estimate the retinal image derived by % the particular luminance inputs. It requires the use of the Image Processing toolbox.

% Attention: large input scenes may need a lot of time to be processed!

%

%--------------------------------------------------------------------------

% INPUTS

% parameters: structure of the set of model parameters defined in CIE standard

% parameters.age : age of the observer in years

% parameters.pigmentationFactor : 0 for very dark eyes,

% 0.5 for brown eyes, 1.0 for blue-green caucasians, up to 1.2 for blue eyes.

% parameters.pixelSize : in mm

% parameters.viewingDistance : in mm

% parameters.range : range in log units for displaying the output

% parameters.verbose : true/false displaying more information

%

% sceneLuminance (double): linear calibrated scene luminance array

% normalized in the range [0,1]

%

%--------------------------------------------------------------------------

% OUTPUTS

% retinalContrast (double): linear retinal contrast array

%

%-------------------------------------------------Calculating filter kernel

% According to equation (8) of Vos and van den Berg (1999) CIE standard

% The glare spread function from the paper is used in order to create a 2D convolution kernel. After that, the %kernel is convolved with the input luminance image in order to estimate the cumulated contributions

% of different points on the scene, to the retinal image.

if (parameters.verbose)

fprintf('1. Calculating filterKernel...\n');

end

radius = max(size(sceneLuminance));

filterKernel = double(zeros((2*radius) + 1));

for i = 1:(2*radius + 1)

for j = 1:(2*radius + 1)

dist = parameters.pixelSize*sqrt((i - (radius + 1))^2 + (j - (radius + 1))^2);

th = atand(dist/parameters.viewingDistance); %glare angle theta

filterKernel(i,j) = (1 - 0.008*(parameters.age/70)^4) * ...

(9.2e6/(1 + (th/0.0046)^2).^1.5 + ...

1.5e5/(1 + (th/0.045)^2).^1.5) + ...

(1 + 1.6*(parameters.age/70)^4) * ...

((400/(1 + (th/0.1).^2) + 3e-8*th^2) + ...

parameters.pigmentationFactor*(1300/(1 + (th/0.1)^2)^1.5 + ...

0.8/(1 + (th/0.1)^2)^0.5)) + ...

2.5e-3*parameters.pigmentationFactor;

filterKernel(i,j)=filterKernel(i,j)*cosd(th);% correction for flat target instead of sphere

end

end

% Normalization of the filter kernel

% The sum of all elements of the filter kernel should sum up to 1, in order

% not to add any DC constant during convolution.

filterKernel=filterKernel./sum(filterKernel(:));

%-------------------------------------------Performing the actual filtering

if (parameters.verbose)

fprintf('2. Filtering (this may take time for large maps)...\n');

end

% Convolution using imfilter function (requires Image Processing toolbox).

% This function minimizes the impact of boundary conditions by replicating

% the border values of the input luminance image. It is also FFT-based, so

% it is faster compared to a typical convolution.

retinalContrast=imfilter(sceneLuminance,filterKernel,'replicate');

%statistics of the retinal contrast image

maxRetinalContrast = max(max(retinalContrast));

minRetinalContrast = min(min(retinalContrast));

rangeRetinalContrast = maxRetinalContrast/minRetinalContrast;

meanRetinalContrast = mean(mean(retinalContrast));

if (parameters.verbose)

fprintf('\n Retinal contrast statistics');

fprintf(['\n max=' num2str(maxRetinalContrast)]);

fprintf(['\n min=' num2str(minRetinalContrast)]);

fprintf(['\n mean=' num2str(meanRetinalContrast)]);

fprintf(['\n range=' num2str(rangeRetinalContrast)]);

fprintf('\n\n');

end

**function [ imageLogDisplay ] = visualizeLogImage( imageLog, logRange )**

%Preprocessing for displaying a logarithmic image

%

%--------------------------------------------------------------------------

% INPUTS

%% imageLog (double): logarithmic (log10) encoding of a [0,1] image. Since the original image is in the interval [0,1], its logarithmic encoding (imageLog) is in the interval (-inf,0].

%

% logRange (double): range (in log units) that will be applied on the %visualization output

%

%--------------------------------------------------------------------------

% OUTPUTS

%

% imageLogDisplay (uint8): visualization output in the interval [0,255] of % the logarithmic input image

n

imageLogDisplay=imageLog;

imageLogDisplay(imageLogDisplay-logRange)=-logRange;%truncate anything below the output log range [-logRange,0]

imageLogDisplay=imageLogDisplay+logRange; %[0,logRange]

imageLogDisplay=imageLogDisplay./logRange; %[0,1]

imageLogDisplay=uint8(imageLogDisplay.*255);%[0,255]

end
